# The Association of IgE Levels with *ADAM33* Genetic Polymorphisms among Asthmatic Patients

**DOI:** 10.3390/jpm11050329

**Published:** 2021-04-22

**Authors:** Malek Zihlif, Amer Imraish, Baeth Al-Rawashdeh, Aya Qteish, Raihan Husami, Rawand Husami, Farah Tahboub, Yazun Jarrar, Su-Jun Lee

**Affiliations:** 1 Department of Pharmacology, Faculty of Medicine, The University of Jordan, Amman 11942, Jordan; fara7tahboub@gmail.com; 2 Department of Biology, Faculty of Science, The University of Jordan, Amman 11942, Jordan; a.imraish@ju.edu.jo; 3 Department of Special Surgery, Faculty of Medicine, The University of Jordan, Amman 11942, Jordan; drbraw@gmail.com (B.A.-R.); aya.qteish@gmail.com (A.Q.); raihan555@hotmail.com (R.H.); rawandhusami13@gmail.com (R.H.); 4 Department of Pharmacy, Al-Zaytoonah University of Jordan, Amman 11733, Jordan; yazun.jarrar@zuj.edu.jo; 5 Department of Pharmacology, College of Medicine, Inje University, Busan 50834, Korea; 2sujun@inje.ac.kr

**Keywords:** asthma, *ADAM33*, IgE, single-nucleotide polymorphism

## Abstract

Total serum immunoglobulin E (IgE) is elevated in multiple allergic diseases and is considered a good predictor of atopy. Several studies have been performed on the association of IgE levels with the polymorphism of the *ADAM33* gene in asthmatic patients. The aim of this study was to determine whether there is an association between IgE levels and the genetic polymorphisms of the *ADAM33* gene (T1, T2, T + 1, V4, S1, S2, and Q-1) in both healthy and asthmatic patients among Jordanians. The clinical data were collected for this case–control study from 267 asthmatic patients and 225 control subjects. Seven genetic polymorphisms (T1, T2, T + 1, V4, S1, S2, and Q-1) of the gene *ADAM33* were analyzed using the polymerase chain reaction/restriction fragment length polymorphism method. The minor alleles (G) of T1, (A) of T2, T + 1, and (G) of V4 polymorphisms were associated with a significant increase in total serum IgE levels in adults but not children. The V4 genetic polymorphism, however, showed a significant association with IgE levels in both adults and children. The S1 polymorphism was significantly associated with the codominant module only in the adults. The S2 polymorphism showed a significant association (*p*-value < 0.05) in both codominant and recessive models. However, in the dominant model for both pediatric control and asthmatic patients, the association between the IgE and S2 polymorphism was insignificant (*p*-value = 0.7271 and 0.5259, respectively). This study found a statistically significant association between multiple *ADAM33* genetic polymorphisms and IgE levels. Such findings add to the growing evidence that the *ADAM33* gene has a major impact on IgE levels among asthmatic patients of Jordanian origin.

## 1. Introduction

Asthma is a chronic respiratory disease characterized by variable levels of airway obstruction and a significant reduction in pulmonary function [1]. It differs in its etiology, severity, and pathogenicity. Although the exact cause and mechanism of asthma are not well understood, it has been proven that there are both genetic and environmental components to the disease [1]. Though asthma is considered a childhood disease, it can also cause significant morbidity and mortality throughout adulthood. The prevalence of asthma has been increasing in the past few decades. Although the cause of such an increase is not well understood, it is thought to be related to the “western lifestyle” [2].

Further investigation of the genetic predisposition of asthma is of great importance to allow for early diagnosis and targeted gene therapy [3], therefore potentially slowing the progression of the disease and improving the quality of life. Numerous genes have been linked to the pathological process of asthma [3], with single-nucleotide polymorphisms (SNPs) of the *ADAM33* gene being one of the most considerably reviewed [4].

The *ADAM33* gene, which codes for zinc metalloproteinases, is located on chromosome 20p13 and is a member of the ADAM family of proteins [5]. The *ADAM33* gene contains 22 exons and 21 introns and encodes 8 domains: A signal sequence, pre-domain, catalytic domain, disintegrin domain, cysteine-rich domain, EGF domain, transmembrane domain, and cytoplasmic domain [6]. These functionally diverse domains are translated into a variety of biological functions that modulate cell activation, proteolysis, adhesion, fusion, and signaling [7]. Over 100 SNPs of the *ADAM33* gene have been correlated with asthma [8]. The relationship between this gene and airway hyperresponsiveness, a major component of asthma, is related to the high level of *ADAM33* gene expression in subepithelial fibroblasts and smooth muscle tissue of the lung [9].

Total serum immunoglobulin E (IgE) is elevated in multiple allergic diseases, such as allergic asthma, and is considered a good predictor of atopy. Many epidemiological studies have demonstrated a strong association between total serum IgE levels and asthma [10]. IgE plays a crucial role in the immune pathophysiology of allergic asthma. Thus, IgE has been used as a target for the management and better control of asthma [11].

Several studies have investigated the association between IgE levels and the polymorphisms of the *ADAM33* gene in asthmatic patients. Yang KD et al, concluded that there is indeed a significant association between IgE levels and several genes, including *ADAM33* [12]. A similar study carried out by Benjamin A. Raby et al. highlighted that a common SNP in *ADAM33*, which is also linked to the increased levels of IgE in asthmatics, was associated with asthma [13]. Furthermore, a study carried out in Iraq by AL-Zubaidy et al. showed that there was a significant increase in IgE levels in relation to the SNPs in *ADAM33* gene [9]. The association between *ADAM33* SNPs and IgE levels was also confirmed in a study performed by Timothy D.Howard et al., that showed a clear association between IgE levels and various SNPs among different races [14]. A study in the Puerto and Mexican population, on the other hand, found no association between *ADAM33* SNPs and IgE levels [15]. Godava et al, also found that none of the 11 SNPs of the *ADAM33* studied were associated with elevated IgE levels or asthma [16]. 

The aim of this study was to determine whether there is an association between IgE levels and the genetic polymorphisms of the *ADAM33* gene (T1, T2, T + 1, V4, S1, S2, and Q-1) in asthma patients of Jordanians. The seven SNPs included in this study were chosen because these SNPs have been previously studied and several of them have been associated with asthma, allergic rhinitis, and/or with increased total IgE levels [16]. To the best of our knowledge and after searching PubMed, Web of Science, and Embase, this association has not been studied among Jordanian asthmatic patients. Further investigation of this relationship is necessary and of great significance to broaden and deepen our understanding of the pathogenesis of asthma to better identify appropriate prevention and treatment strategies.

## 2. Materials and Methods

### 2.1. Populations

This case–control study encompassed 107 pediatric asthmatic patients, 115 healthy pediatric controls, 160 adult asthmatic patients, and 110 healthy adult controls. In addition, 95 samples were collected from adult allergic rhinitis patients. The chief inclusion criterion for all groups was ethnicity. Participants were exclusively drawn from the same ethnic group (namely Arabs) in order to minimize population stratification. Ethnicity was determined by self-report. The Institutional Review Board at the University of Jordan approved the study protocol (reference number 41/2009/IRB L). The control subjects and the patients were enrolled for the study at the University of Jordan Hospital and Jordan Hospital in Amman, Jordan. All control subjects were asymptomatic and had no history of asthma or other atopic disorders. Diagnosis of asthma in children was made through a detailed history and physical examination conducted by the treating physician. Adults underwent pulmonary function tests to support the diagnosis of asthma in addition to the history and physical examination by the treating physician. Children did not undergo pulmonary function tests as the use of spirometry is generally infeasible for patients under the age of 5 years [17]. The sample size of 267 patients was representative of the total asthmatic patients (*n* = 800) attending the University of Jordan Hospital. The sample size was calculated using the power of test 1 − β = 0.8, 5% margin of error, and 95% confidence level. Furthermore, for further validating our hypothesis, we included other 95 patients suffering from allergic rhinitis. 

### 2.2. Total Immunoglobulin E Level Measurement 

Total serum immunoglobulin E (IgE) levels were measured using a Total IgE ElISA kit (IBL Inter-national). Results were read using ELISA Reader. Corresponding to the kit ranges, subjects with IgE levels below 100 IU/mL were considered as having normal IgE levels [18]. 

### 2.3. DNA Extraction 

Genomic DNA was extracted using the Wizard Genomic DNA purification Kit (Promega) from 5 mL peripheral blood samples that were collected in EDTA tubes. Isolated DNA was stored at −20 °C until further use. 

### 2.4. Genotyping of ADAM33 Gene by PCR-RFLP

The genetic polymorphism analysis of 7 SNPs for the *ADAM33* gene was determined by using the polymerase chain reaction (PCR)/restriction fragment length polymorphism method. The primers and restriction enzymes were similar to those used in our previous publication [19]. The PCR products were digested by restriction endonucleases overnight, which were then subsequently separated by electrophoresis on agarose gel and stained with ethidium bromide. In total, 7 SNPs were tested: T1, T2, T + 1, V4, S1, S2, and Q-1. 

### 2.5. Linkage Disequilibrium and Haplotype Distribution

The linkage disequilibrium (LD) analysis, estimation of haplotype diversity, and deviation from Hardy–Weinberg equilibrium were carried out using Haploview 4.2 software. The LD for each pair of genetic variants was measured using |D′| and correlation coefficient (r^2^).

### 2.6. Statistical Analysis

All statistics were calculated using GraphPad Prism 7.0 software (GraphPad Software, La Jolla, CA). The significant departure from the Hardy–Weinberg equilibrium (HWE) was assessed by the procedure described by Guo and Thompson, using a test analogous to Fisher’s exact test as implemented in ARLEQUIN (Excoffier et al., 2005). Data obtained from this study passed the normality test for all the groups and differences were compared using parametric tests. Data are represented as mean ± S.E.M. The power of the tests was estimated using a difference of IgE levels among asthmatic patients according to *ADAM33* genotype, with a 2-sided significance level of 0.05. The power of the test ranged from 0.69 to 0.92 for the different genotypes. Differences were compared using student’s *t*-test for dominant and recessive models and 1-way analysis of variance (ANOVA) for goodness-of-fit, followed by Tukey’s multiple comparison test for the codominant model. The criterion for statistical significance was *p* < 0.05.

## 3. Results

The study had a total of 148 pediatric participants, of which 49 pediatric asthmatics had an average age of 5.96 ± 4.63, while the 99 pediatric control subjects had an average age of 7.53 ± 4.83 (Table 1). In total, there were 261 adult participants, which included 151 asthmatic adults with an average age of 44.73 ± 15.41 and 110 healthy adults with an average age was 38.74 ± 14.85 (Table 1). The demographic data of the allergic rhinitis are presented in Supplementary Table S1. All subjects were included in the association statistics and dropout was recorded. The allele frequencies of the *ADAM33* gene polymorphisms in patients and healthy controls are shown in Table 2 and Table 3. All SNPs were in HWE, as the null hypothesis for departure from HWE was rejected for all SNPs (Table 2 and Table 3). No significant differences in the allelic frequencies of all SNPs tested between asthmatic patients and the control volunteers were found. Table 4 shows the results of the association between seven SNPs for the *ADAM33* gene and total serum IgE levels in both healthy and asthmatic adult and child participants. 

The minor allele (G) of the T1 allele and (A) of the T2 allele were associated with a significant increase in total serum IgE levels in adults. In the case of children, the T1 and T2 alleles showed an insignificant association with IgE levels (*p*-value > 0.05), despite an increase in IgE levels in the participants homozygous for mutant (GG) and (AA). The associations of the T1 and T2 SNPs with IgE levels were significant for the dominant model in adults (*p* = 0.0047 and 0.0038, respectively), while in children, they showed a tendency toward significance with *p* = 0.0806 and 0.0729, respectively. Alternatively, the T2 allele showed a significant association in the recessive model in both adults and children (*p* = 0.0467 and 0.0071, respectively). However, such an association was insignificant (*p*-value > 0.05) in the case of T1 in both adults and children. In the case of the T + 1 allele, significant associations were found in the dominant model in both adults and children with *p*-values of 0.0029 and 0.0291, respectively. In addition, the T + 1 allele showed a significant association in the recessive model in children (*p* = 0.0093). However, this association was insignificant in adults (*p* = 0.1888).

The minor allele (G) of V4 was significantly (*p*-value < 0.05) associated with decreased total serum IgE levels in both adults and children. The V4 allele showed a significant association with IgE levels in the recessive model in both adults and children (*p* = 0.0001). V4 also exhibited a significant association in the dominant model in children (*p* = 0.0345). However, such an association was not found in adults (*p* = 0.0768). 

The S1 allele showed a significant association in the codominant model, with decreased IgE levels in the minor allele (AA) in adults only. In terms of dominant and recessive models, the S1 allele in adults exhibited a significant association with a *p*-value = 0.0001. Those models were not applicable in the case of the S1 allele in children, as S1 is made only of two genotypes: (GG and GA). 

Regarding the Q-1 SNP, the minor allele (C) was associated with a decrease in total serum IgE levels in adults and children. The Q-1 allele also showed a significant association in the dominant model (*p* = 0.0013) in adults. However, such an association was insignificant in children (*p* = 0.0875). Additionally, the Q-1 allele showed a significant association in the recessive model in children (*p* = 0.0001). However, such an association was not found in the case of adults (*p* = 0.6842).

Table 5 lists the results of the analysis of the association between SNPs in *ADAM33* and total serum IgE levels. Among adults, the minor allele (G) of T1 and (A) of T2 were associated with a statistically significant increase in IgE levels in asthmatic patients compared with controls. Under both dominant (*p* = 0.024, 0.0175, respectively) and recessive (*p* = 0.0407, 0.015, respectively) models, there was a significant association with IgE levels. As for the control participants, there was a statistically significant association in the recessive model. However, in case of the dominant model, the association was not statistically significant (*p*-value > 0.05) for both T1 and T2 alleles. Regarding asthma patients, the T + 1 genotype showed a significant association (*p*-value < 0.05) in both codominant and dominant models with a strong association between the increased IgE levels and the minor allele (A). However, in the control participants, the recessive model exhibited a significant association (*p* = 0.0188) only and did not show a significant association in the dominant model (*p* = 0.3069). 

In the case of V4, there was a statistically significant association in the recessive model in both controls and asthmatic patients (*p* = 0.0494 and 0.0236, respectively). However, in the dominant model, the association with IgE levels did not reach statistical significance in controls or asthmatic patients (*p* = 0.115 and 0.8174, respectively). The statistically significant associations were identified in the S1 allele case in asthmatic patients for both dominant and recessive models, with a *p*-value of 0.0001 for both models. The S1 genotype also showed a significant association in the codominant model, in which the IgE levels decreased in the minor allele (A). There was no significant association (*p*-value > 0.05) between the IgE levels and the S2 and Q-1 genetic polymorphisms in all the genotype models in both controls and asthma patients. 

In the case of children, there was no association (*p*-value > 0.05) between the T1 allele and total levels of serum IgE in both controls and asthmatic patients for the codominant and recessive models. On the contrary, there was a statistically significant relationship between serum IgE levels and the T1 SNP in the dominant model for the control group (*p* = 0.0179). However, this relation was insignificant in asthmatic children (Table 6). As demonstrated in Table 6, the serum IgE levels did not show any association with the genotype of T2 and T + 1 genetic polymorphisms in all three models in both the control groups and asthmatic children. Among children, the minor allele (G) of the V4 SNP was associated with decreased IgE levels in asthmatic children compared with the control group. Under the recessive model, there was a significant association with IgE levels in both the control group and asthmatic children (*p* = 0.0430, 0.0168, respectively). The S1 genotype in children was distributed into only two genotypes (GG and GA) that were not associated with serum IgE levels. The S2 genotype showed a significant association (*p* < 0.05) with asthma risk in both codominant and recessive models in asthmatic children. In contrast, the association between the IgE level and S2 genotype was insignificant in the dominant model in both the control group and asthmatic children (*p* = 0.7217 and 0.5259, respectively). The Q-1 genotype was found to be associated with the control group in the dominant and codominant models. However, such an association showed a tendency toward significance with *p* = 0.0829 in the recessive model.

For further validating our hypothesis, we tested the association of the *ADAM33* genotype with allergic rhinitis. We found that all tested *ADMA33* genetic variants except S2 were significantly associated (*p*-value < 0.05) with allergic rhinitis using the codominant and recessive models (Supplementary Table S2). 

We also found that *ADAM33* T1, T2, and T + 1 genotypes were significantly associated with the IgE levels among allergic rhinitis patients (Supplementary Table S3). The homozygous T1, T2, and T + 1 genotypes had significantly (*p*-value < 0.05) lower IgE levels than the wild and heterozygous genotypes.

Regarding the frequency of the *ADAM33* haplotype, we found that the T1 (A), T2 (G), T + 1 (G), V4 (C), S1 (C), S2 (G), Q-1 (G) *ADAM33* haplotype was the most common haplotype among adults and children healthy and asthma subjects (Table 7). However, the frequency of the T1 (A), T2 (G), T + 1 (G), V4 (C), S1 (C), S2 (G), Q-1 (G) *ADAM33* haplotype was significantly (X^2^, *p*-value < 0.05) lower among asthmatic subjects than healthy subjects in both children and adults. In addition, the T1 (A), T2 (G), T + 1 (G), V4 (C), S1 (C), S2 (C), Q-1 (A) *ADAM33* haplotype frequency was significantly (*p*-value < 0.05) higher among adult asthmatic patients (0.12) than healthy controls (0.07). Furthermore, we found the T1 (G), T2 (A), T + 1 (A), V4 (G), S1 (C), S2 (G), Q-1 (A) *ADAM33* haplotype among only asthmatic children (0.04), and this haplotype was not detected among other groups in this study (Table 7). 

**Figure 1:**
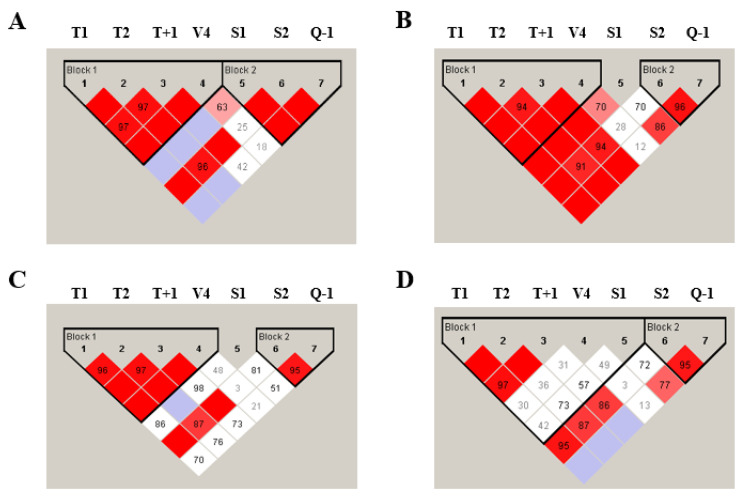
Linkage disequilibrium (LD) of *ADAM33* genetic variants found among healthy and asthmatic subjects. The LD of healthy adults (**A**), asthma adults (**B**), healthy children (**C**), and asthma children (**D**) was carried out using Haploview software. The red squares represent a strong LD and the white squares represent a weak LD. The blue squares indicate that there is no LD.

The LD of the studied *ADAM33* genetic variants is shown on Figure 1. We noticed that the *ADAM33* S1 variant was in complete LD (D’) with the T1, T2, T + 1, and V4 variants among asthma adults (Figure 1B). Indeed, the LD between S1 and other *ADAM33* genetic variants was weak in healthy adult controls (Figure 1A). In addition, the LD between Q-1 and T1 and T2 was higher among asthma than healthy adult subjects (Figure 1A,B). Among children, the V4 variant was in complete LD (D’) with T1, T2, and T + 1 variants in healthy subjects, while the LD between V4 and other variants was low among asthmatic patients. 

## 4. Discussion

In this study, we examined the association of IgE levels with the polymorphisms of the *ADAM33* gene specifically, 7 SNPs (T1, T2, T + 1, V4, S1, S2, and Q-1) among asthmatic Jordanian patients. This is the first study to illustrate the relationship between *ADAM33* SNPs and IGE levels among the Jordanian population. Such study will shed the light on the role of genes such as *ADAM33* and IGE levels. There was a significant association between various genotypes of *ADAM33* genetic polymorphisms and the IgE levels among children and adults. IgE is an antibody produced by the immune system in response to allergens that the body may be exposed to. Interestingly, many of the studied *ADAM33* genetic polymorphisms have been found to be associated with IgE levels in either adult or pediatric cohort studies. In the case of the T1 and T2 polymorphisms, the minor allele (G) of T1 and (A) of T2 were significantly associated with increased total serum IgE levels in adults. However, such an association was insignificant in children, although there was an increase in IgE levels in subjects who had the homozygous mutant (GG) and (AA). These findings are in agreement with a study conducted in a population of the Caribbean coast of Colombia that showed a significant association of the (GG) genotype of the T1 polymorphism with high total IgE levels (*p* = 0.04) and the (AA) genotype of T2 with high total IgE (*p* = 0.02) and high levels of IgE against *Dermatophagoides pteronyssinus* (*p* = 0.03) [20]. A similar result was also observed in a study carried out on Egyptian children that reported significantly higher IgE levels in patients with T1 (GG) genotype [21]. In asthmatic adults, we identified a significant association of T1 and T2 with IgE levels in both dominant (*p* = 0.024 and 0.0175, respectively) and recessive models (*p* = 0.0407 and 0.015, respectively). Such an association was found in the control subjects in the recessive model only. Similarly, a study conducted in four populations, namely African American, white American, Hispanic, and white Dutch, showed a significant association between serum IgE levels with *ADAM33* T1 and T2 genetic polymorphisms in both the white American (*p* = 0.0033 and 0.0037, respectively) and Hispanic subjects (*p* = 0.0099 and 0.0092) [16]. However, there was no association between T1 polymorphism and log-transformed total IgE levels in the Korean population [22]. In children, no significant association was found between total IgE levels and T1 and T2 polymorphisms in both controls and cases in all three models except in the dominant model for the control group in the case of the T1 polymorphism (*p* = 0.017). Similar results emerged from studies conducted in other ethnicities, where no significant association was observed between the T1 and T2 polymorphisms and IgE levels among both the Mexican or Puerto Rican populations or between T2 and IgE levels in Egyptian children [14,20]. Godava et al, also concluded that none of the polymorphisms were associated with elevated IgE levels or asthma [16]. On the other hand, the T1 polymorphism was significantly associated with serum IgE levels among Hispanics in a North American population of childhood asthmatic patients (*p* = 0.04) [13].

We also observed that the T + 1 polymorphism was significantly associated with IgE levels in both dominant and recessive models in adults. Such an association was observed in the dominant model but not in the recessive. Comparing the association of the T + 1 polymorphism with serum IgE levels among asthmatic and healthy adult subjects revealed significant associations with the codominant and dominant models in both cases and with the recessive model in healthy subjects. In children, no significant association was observed in all three models in both healthy and asthmatic subjects. A study performed by Godava et al, showed no significant association between T + 1 and the other 10 genetic polymorphisms in the *ADAMS33* gene with serum IgE levels [15]. In contrast, the association was significant among Hispanics (*p* = 0.04) [13].

Regarding the V4 polymorphism, a significant association in the recessive model in both adults and children was found (*p* = 0.0001) (Table 1). However, a significant association was found in the dominant model in children (*p* = 0.0345), but not for adults (*p* = 0.0768). A study conducted in Korea [22] which examined five genetic polymorphisms that were originally reported to be associated with asthma and IgE levels showed that there was no associated genetic polymorphism with log-transformed IgE levels. Studies conducted in China [23] and the U.S. [24] also found a significant association between the *ADAM33* V4 allele and asthma (*p* = 0.0001 and *p* = 0.032, respectively). In correlation with these results, a study in Egypt [21] which examined T1, T2, and V4 alleles showed no association between V4 and IgE levels. Interestingly, the V4 polymorphism showed a positive correlation, with passive smoking being a precipitating factor and showing borderline significance with absolute eosinophilia. A study conducted in Columbia [20] examined the association of the *ADAM33* polymorphisms with asthma, particularly mite-specific IgE levels in the population of Caribbean coast of Colombia. This modification distinguished the Colombian study form the rest. The authors found that the V4 polymorphism was significantly associated with IgE levels against *B. tropicalis* (*p* = 0.03). Among children, the minor allele (G) of V4 was correlated with reduced IgE levels in asthmatic children compared with controls. As shown in the recessive model, there was a significant association with IgE levels in both healthy and asthmatic children (*p* = 0.0430, 0.0168, respectively). A known study conducted in the Mexican and Puerto Rican populations showed no association between V4 polymorphism and IgE levels and asthma [14]. 

In case of the S1, S2, and Q-1 alleles, the results were mixed. The S1 allele showed a significant association in the codominant module only for adults, in which the IgE levels decreased in the minor allele (A). However, a study [22] conducted in Korea showed no association with the S1 polymorphism and IgE levels. The S2 polymorphism showed a significant association (*p*-value < 0.05) in both the codominant and recessive models. On the contrary, in the dominant model for both pediatric control and asthmatic patients, the association between the IgE and S2 SNP was shown to be insignificant (*p*-value = 0.7271 and 0.5259, respectively). In a study conducted in the Netherlands, a significant association was demonstrated between the S2 SNP and total serum IgE levels in an asthma-related phenotype in the Dutch and U.S. white population. Significant associations were observed in the codominant model (*p*-value = 0.014) and recessive model (*p*-value = 0.031), respectively [16]. 

In the current study, the Q-1 allele demonstrated a significant association in the dominant model (*p* = 0.0013) in adults. Nonetheless, this was not seen in children (*p* = 0.0875). Furthermore, a significant association in the recessive model in children (*p* = 0.0001) was seen. However, this was not seen in adults (*p* = 0.6842). 

In order to validate our hypothesis that *ADAM33* genetic variants are associated pulmonary allergic diseases, we included more 95 genomic sample from allergic rhinitis patients. We found that the *ADAM33* genotype is strongly associated with the allergic rhinitis. Our results are in line with what was reported previously that *ADAM33* gene is associated with allergic rhinitis patients of Asian [24] and Caucasian [25] origin. 

Regarding the relationship between *ADAM33* and the IgE protein, evidence has shown a significant role for *ADAM33* in intercellular adhesion. *ADAM33* has a disintegrin domain that supports α9β1 integrin-dependent leukocyte adhesion and expression of integrin in mesenchymal cells, such as fibroblasts and smooth muscle cells [26]. Thus, SNPs in the *ADAM33* disintegrin domain function in intercellular adhesion and may therefore partially influence the quantity of leukocytes and the inflammation reaction itself in the airways, which usually reflects on the IgE [27].

## 5. Conclusions

In conclusion, the findings of this study found a significant association between multiple *ADAM33* genetic polymorphisms and IgE levels, a major component in the pathophysiology of asthma. Such finding may help in clinical diagnosis by better defying asthma sub-phenotypes. It also approves the risk value of *ADAM33* in IgE-dependent diseases such as atopic asthma and allergic rhinitis. To date and to the best of our knowledge, this is the first study that has examined the association of IgE levels with the genetic polymorphisms of *ADAM33* gene among Jordanian asthmatic patients.

## Figures and Tables

**Table 1:** Characteristics of the study population.

Phenotype	Sample Size	% Female	% Male	Av. Age ± SD
Adult asthma	151	0.71	0.29	44.73 ± 15.41
Adult control	110	0.47	0.53	38.74 ± 14.85
Children asthma	99	0.31	0.69	5.96 ± 4.63
Children control	49	0.38	0.62	7.53 ± 4.83

**Table 2:** Minor allele frequencies and their association analyses in adults.

SNP Name	Nucleotide Change	Total Frequency	Frequency in Control	Frequency in Asthma	HWE Exact *p*
T1	A/G	0.2	0.18	0.22	0.45
T2	G/A	0.21	0.19	0.23	0.36
T + 1	G/A	0.21	0.18	0.23	0.72
V4	C/G	0.2	0.19	0.2	0.68
S1	G/A	0.06	0.05	0.06	0.6
S2	C/G	0.37	0.32	0.41	0.9
Q-1	G/A	0.16	0.12	0.19	0.073

**Table 3:** Minor allele frequencies and their association analyses in children.

SNP Name	Nucleotide Change	Total Frequency	Frequency in Control	Frequency in Asthma	HWE Exact *p*
T1	A/G	0.19	0.19	0.2	0.81
T2	G/A	0.22	0.22	0.22	0.85
T + 1	G/A	0.2	0.2	0.21	0.22
V4	C/G	0.25	0.2	0.31	0.049
S1	G/A	0.08	0.07	0.09	0.37
S2	C/G	0.41	0.41	0.41	1.0
Q-1	G/A	0.2	0.2	0.21	0.84

**Table 4:** Comparison between IgE levels of asthma patients and control group according to *ADAM33* SNPs in adults and children.

	Model	Genotype	Adult (*n* = 261) IgE Levels: IU/mL (*n*)	Genotype Comparison (*p*-Value)	Children (*n* = 148) IgE Levels: IU/mL (*n*)	Genotype Comparison (*p*-Value)
T1	Codominant	AA	71.1 ± 3.6 (168)	AA vs. AG (0.0084)	93.0 ± 9.1 (100)	AA vs. AG (0.5109)
		AG	93.1 ± 2.2 (81)	AA vs. GG (0.0235)	51.0 ± 4.8 (41)	AA vs. GG (0.1383)
		GG	127.2 ± 7.8 (12)	AG vs. GG (0.6344)	157.5 ± 2.1 (7)	AG vs. GG (0.3067)
	Dominant	AA		(0.0047)		(0.0806)
		AG + GG				
	Recessive	AA + AG		(0.1447)		(0.1028)
		GG				
T2	Codominant	GG	77.1 ± 6.6 (165)	GG vs. GA (0.0111)	99.6 ± 1.9 (92)	GG vs. GA (0.5906)
		GA	93.3 ± 2.4 (83)	GG vs. AA (0.0064)	49.3 ± 2.1 (49)	GG vs. AA (0.1497)
		AA	139.4 ± 2.8 (13)	GA vs. AA (0.3914)	157.1 ± 1.3 (7)	GA vs. AA (0.2734)
	Dominant	GG		(0.0038)		(0.0729)
		GA + AA				
	Recessive	GG + GA		(0.0467)		(0.0071)
		AA				
T + 1	Codominant	GG	70.6 ± 3.5 (164)	GG vs. GA (0.0044)	98.5 ± 9.47 (98)	GG vs. GA (0.6622)
		GA	92.4 ± 2.62 (84)	GG vs. AA (0.0230)	55.3 ± 3.9 (42)	GG vs. AA (0.1745)
		AA	117.4 ± 2.2 (13)	GA vs. AA (0.7278)	139.2 ± 4.5 (8)	GA vs. AA (0.2734)
	Dominant	GG		(0.0029)		(0.0291)
		GA + AA				
	Recessive	GG + GA		(0.1888)		(0.0093)
		AA				
V4	Codominant	CC	85.3 ± 6.4 (169)	CC vs. CG (0.6329)	86.5 ± 4.1 (93)	CC vs. CG (0.5830)
		CG	93.7 ± 4.7 (80)	CC vs. GG (0.0001)	49.1 ± 7.5 (48)	CC vs. GG (0.0144)
		GG	40.0 ± 1.2 (12)	CG vs. GG (0.0002)	23.2 ± 5.3 (7)	CG vs. GG (0.0920)
	Dominant	CC		(0.0768)		(0.0345)
		CG + GG				
	Recessive	CC + CG		(0.0001)		(0.0001)
		GG				
S1	Codominant	GG	90.1 ± 4.6 (230)	GG vs. GA (0.0001)	85.4 ± 3.2 (124)	GG vs. GA (0.1529)
		GA	26.5 ± 2.5 (28)	GG vs. AA (0.0001)	87.2 ± 4.7 (24)	
		AA	0.8 ± 0.05 (3)	GA vs. AA (0.2503)	None	
	Dominant	GG		(0.0001)	N/A	
		GA + AA				
	Recessive	GG + GA		(0.0001)	N/A	
		AA				
S2	Codominant	CC	98.5 ± 5.5 (103)	CC vs. CG (0.2055)	124.4 ± 7.5 (56)	CC vs. CG (0.9958)
		CG	81.6 ± 6.1 (122)	CC vs. GG (0.0404)	60.7 ± 7.1 (69)	CC vs. GG (0.0923)
		GG	65.1 ± 1.8 (36)	CG vs. GG (0.2936)	43.9 ± 2.9 (23)	CG vs. GG (0.0582)
	Dominant	CC		(0.0963)		(0.2211)
		CG + GG				
	Recessive	CC + CG		(0.1061)		(0.0152)
		GG				
Q-1	Codominant	TT	80.9 ± 3.1 (189)	TT vs. TC (0.0009)	98.0 ± 8.7 (97)	TT vs. TC (0.5724)
		TC	45.5 ± 3.6 (62)	TT vs. CC (0.3344)	67.8 ± 3.3 (36)	
		CC	6.9 ± 3.8 (10)	TC vs. CC (0.5633)	10.8 ± 1.8 (5)	
	Dominant	TT		(0.0013)		(0.0875)
		CT + CC				
	Recessive	TT + CT		(0.6842)		(0.0001)
		CC				

**Table 5:** Comparison between IgE levels of asthma patients and control group according to *ADAM33* SNPs in adults.

	Model	Genotype	Control (*n* = 110) IgE Levels: IU/mL (*n*)	Genotype Comparison (*p*-Value)	Asthma (*n* = 151) IgE Levels: IU/mL (*n*)	Genotype Comparison (*p*-Value)
T1	Codominant	AA	14.9 ± 2.1 (75)	AA vs. AG (0.2664)	116.4 ± 3.6 (93)	AA vs. AG (0.0367)
		AG	25.1 ± 4.8 (31)	AA vs. GG (0.0402)	135.4 ± 4.7 (50)	AA vs. GG (0.0420)
		GG	3.4 ± 1.2 (4)	AG vs. GG (0.1963)	189.1 ± 12.5 (8)	AG vs. GG (0.6116)
	Dominant	AA		(0.0795)		(0.0240)
		AG + GG				
	Recessive	AA + AG		(0.0188)		(0.0407)
		GG				
T2	Codominant	GG	15.3 ± 2.1 (73)	GG vs. GA (0.4522)	126.3 ± 8.9 (92)	GG vs. GA (0.0353)
		GA	23.6 ± 4.6 (33)	GG vs. AA (0.0454)	139.4 ± 4.0 (50)	GG vs. AA (0.0248)
		AA	3.4 ± 1.2 (4)	GA vs. AA (0.1607)	199.9 ± 5.4 (9)	GA vs. AA (0.5096)
	Dominant	GG		(0.1570)		(0.0175)
		GA + AA				
	Recessive	GG + GA		(0.0188)		(0.0150)
		AA				
T + 1	Codominant	GG	15.9 ± 2.2 (74)	GG vs. GA (0.7063)	115.7 ± 2.1 (90)	GG vs. GA (0.0344)
		GA	22.5 ± 4.6 (32)	GG vs. AA (0.0597)	135.5 ± 3.3 (52)	GG vs. AA (0.0779)
		AA	3.4 ± 1.2 (4)	GA vs. AA (0.1359)	168.1 ± 1.4 (9)	GA vs. AA (0.7501)
	Dominant	GG		(0.3069)		(0.0270)
		GA + AA				
	Recessive	GG + GA		(0.0188)		(0.0765)
		AA				
V4	Codominant	CC	17.2 ± 2.5 (75)	CC vs. CG (0.2281)	139.7 ± 8.1 (94)	CC vs. CG (0.9058)
		CG	16.5 ± 3.8 (28)	CC vs. GG (0.0647)	135.4 ± 6.8 (52)	CC vs. GG (0.0339)
		GG	21.3 ± 6.9 (7)	CG vs. GG (0.4108)	66.2 ± 7.76 (5)	CG vs. GG (0.0650)
	Dominant	CC		(0.1150)		(0.8174)
		CG + GG				
	Recessive	CC + CG		(0.0494)		(0.0236)
		GG				
S1	Codominant	GG	17.9 ± 2.2 (99)	GG vs. GA (0.3436)	144.8 ± 4.3 (131)	GG vs. GA (0.001)
		GA	12.0 ± 4.1 (11)		72.8 ± 8.4 (17)	GG vs. AA (0.001)
		AA	None		0.80 ± 0.05 (3)	GA vs. AA (0.6143)
	Dominant	GG	N/A			(0.0001)
		GA + AA				
	Recessive	GG + GA				(0.0001)
		AA				
S2	Codominant	CC	16.2 ± 2.7 (52)	CC vs. CG (0.8768)	182.5 ± 9.09 (51)	CC vs. CG (0.6011)
		CG	16.8 ± 3.0 (45)	CC vs. GG (0.7129)	119.5 ± 4.36 (77)	CC vs. GG (0.7842)
		GG	23.9 ± 4.1 (13)	CG vs. GG (0.4698)	88.5 ± 2.28 (23)	CG vs. GG (0.5157)
	Dominant	CC		(0.5982)		(0.1281)
		CG + GG				
	Recessive	CC + CG		(0.2437)		(0.3601)
		GG				
Q-1	Codominant	TT	17.3 ± 2.3 (84)	TT vs. TC (0.5607)	131.8 ± 4.0 (105)	TT vs. TC (0.0224)
		TC	18.0 ± 4.6 (25)	TT vs. CC (0.2417)	64.1 ± 3.3 (37)	TT vs. CC (0.8103)
		CC	3.7 ± 0.1 (3)	TC vs. CC (0.3069)	8.4 ± 2.0 (7)	TC vs. CC (0.4156)
	Dominant	TT		(0.3717)		(0.1123)
		CT + CC				
	Recessive	TT + CT		(0.2721)		(0.1187)
		CC				

**Table 6:** Comparison between IgE levels of asthma patients and control group according to *ADAM33* SNPs in children.

	Model	Genotype	Control (*n* = 99) IgE Levels: IU/mL (*n*)	Genotype Comparison (*p*-Value)	Asthma (*n* = 49) IgE Levels: IU/mL (*n*)	Genotype Comparison (*p*-Value)
T1	Codominant	AA	19.7 ± 2.7 (68)	AA vs. AG (0.5680)	248.9 ± 2.5 (32)	AA vs. AG (0.5109)
		AG	22.7 ± 4.1 (27)	AA vs. GG (0.9403)	105.5 ± 7.3 (14)	AA vs. GG (0.1383)
		GG	20.6 ± 6.2 (4)	AG vs. GG (0.8742)	340.0 ± 2.3 (3)	AG vs. GG (0.3067)
	Dominant	AA		(0.0179)		(0.2772)
		AG + GG				
	Recessive	AA + AG		(0.4438)		(0.1447)
		GG				
T2	Codominant	GG	20.5 ± 2.9 (61)	GG vs. GA (0.8897)	255.5 ± 5.0 (31)	GG vs. GA (0.5906)
		GA	26.6 ± 5.1 (34)	GG vs. AA (0.3014)	101.6 ± 4.9 (15)	GG vs. AA (0.1497)
		AA	19.9 ± 6.5 (4)	GA vs. AA (0.3881)	340.0 ± 2.3 (3)	GA vs. AA (0.2734)
	Dominant	GG		(0.6909)		(0.3373)
		GA + AA				
	Recessive	GG + GA		(0.2947)		(0.1461)
		AA				
T + 1	Codominant	GG	19.9 ± 2.6 (67)	GG vs. GA (0.4440)	268.4 ± 0.47 (31)	GG vs. GA (0.6622)
		GA	29.6 ± 6.2 (27)	GG vs. AA (0.3902)	101.6 ± 4.9 (15)	GG vs. AA (0.1745)
		AA	18.83 ± 5.1 (5)	GA vs. AA (0.7032)	340.0 ± 2.3 (3)	GA vs. AA (0.2734)
	Dominant	GG		(0.3517)		(0.3975)
		GA + AA				
	Recessive	GG + GA		(0.4544)		(0.1632)
		AA				
V4	Codominant	CC	25.6 ± 3.5 (62)	CC vs. CG (0.1723)	208.4 ± 1.7 (31)	CC vs. CG (0.5830)
		CG	16.6 ± 3.1 (34)	CC vs. GG (0.0164)	128.1 ± 5.6 (14)	CC vs. GG (0.0144)
		GG	22.6 ± 8.5 (3)	CG vs. GG (0.1809)	23.7 ± 7.9 (4)	CG vs. GG (0.0920)
	Dominant	CC		(0.0839)		(0.1709)
		CG + GG				
	Recessive	CC + CG		(0.0430)		(0.0168)
		GG				
S1	Codominant	GG	23.5 ± 2.8 (85)	GG vs. GA (0.2337)	220.5 ± 9.3 (39)	GG vs. GA (0.1529)
		GA	16.3 ± 4.7 (14)		186.5 ± 3.9 (10)	
		AA	None		None	
	Dominant	GG	N/A		N/A	
		GA + AA				
	Recessive	GG + GA	N/A		N/A	
		AA				
S2	Codominant	CC	23.6 ± 4.0 (35)	CC vs. CG (0.2847)	292.4 ± 3.5 (21)	CC vs. CG (0.9958)
		CG	22.2 ± 4.2 (47)	CC vs. GG (0.3025)	143.1 ± 9.2 (22)	CC vs. GG (0.0923)
		GG	20.9 ± 3.8 (17)	CG vs. GG (0.0630)	109.1 ± 9.2 (6)	CG vs. GG (0.0582)
	Dominant	CC		(0.7217)		(0.5259)
		CG + GG				
	Recessive	CC + CG		(0.0893)		(0.0444)
		GG				
Q-1	Codominant	TT	25.1 ± 3.4 (64)	TT vs. TC (0.0339)	239.4 ± 9.3 (33)	TT vs. TC (0.5724)
		TC	18.7 ± 3.6 (30)	TT vs. CC (0.0176)	160.1 ± 1.4 (16)	
		CC	10.8 ± 1.8 (5)	TC vs. CC (0.4316)		
	Dominant	TT		(0.0127)		N/A
		CT + CC				
	Recessive	TT + CT		(0.0829)		N/A
		CC				

**Table 7:** The frequency of the *ADAM33* haplotype among adult and children asthma and rhinitis patients.

*ADAM33* Haplotype							Frequency			
T1	T2	T + 1	V4	S1	S2	Q-1	Healthy Adults	Asthma Adults	Healthy Children	Asthma Children
A	G	G	C	C	G	G	0.53	0.43 *	0.49	0.42 #
G	A	A	C	C	C	G	0.18	0.22	0.17	0.16
A	G	G	G	C	G	G	0.14	0.15	0.10	0.15
A	G	G	C	C	C	A	0.07	0.12 *	0.09	0.09
A	G	G	G	T	C	A	0.04	0.05	0.03	0.04
A	G	G	C	C	C	A	0.02	0	0.01	0
A	G	G	C	T	C	A	0.02	0	0.02	0.03
A	G	G	G	C	C	A	0	0.02	0.05	0.04
A	G	G	G	T	C	G	0	0	0.01	0
G	A	A	C	C	C	A	0	0	0.02	0
G	A	A	G	C	G	A	0	0	0	0.04 #
A	G	G	G	T	G	G	0	0	0	0.02

“*” indicates statistical significance in comparison with the adult healthy subjects, while “#” indicates statistical significance in comparison to healthy children subjects.

## Data Availability

Data available on request from the corresponding author.

## Supplementary Materials

The following are available online at https://www.mdpi.com/article/10.3390/jpm11050329/s1, Table S1: The demographic data of allergic rhinitis patients, Table S2: The demographic data of allergic rhinitis patients, Table S3: The association of *ADAM33* genetic variants with the IgE levels among allergic rhinitis patients.
