# Supplementary material for: The Association of IgE Levels with ADAM33 Genetic Polymorphisms among Asthmatic Patients
Source: J Pers Med. 2021 Apr 22;11(5):329. doi: 10.3390/jpm11050329 (PMC8146671; doi:10.3390/jpm11050329)
Supplement: Supplementary file 1 [file jpm-11-00329-s001.zip › jpm-1032652-supplementary.pdf]

Supplementary Table S1. The demographic data of allergic rhinitis patients

Phenotype	Sample size	% female	% male	Av. age ±SD
**Allergic rhinitis**	95	62	38	30.91±15.41

Supplementary Table S2. The allele and genotype frequency of *ADAM33* genetic variants among allergic rhinitis patients

	Model	Genotype	Allergic rhinitis (n=95) IgE levels: IU/ml (n)	Genotype comparison (P-value)
T1	Co-dominant	AA	71.1±5.1 (51)	AA *vs* AG (0.8648)
		AG	117.9±3.1 (40)	AA *vs* GG (0.0002)
		GG	25.1±8.4 (4)	AG *vs* GG (0.0014)
	Dominant	AA		(0.9587)
		AG+GG		
	Recessive	AA+AG		(<0.0001)
		GG		
	Variant allele frequency		0.25	
T2	Co-dominant	GG	73.1±5.6 (49)	GG *vs* GA (0.9757)
		GA	113.3±9.8 (42)	GG *vs* AA (0.0002)
		AA	25.1±8.4 (4)	GA *vs* AA (0.001)
	Dominant	GG		(0.8098)
		GA+AA		
	Recessive	GG+GA		(<0.0001)
		AA		
	Variant allele frequency		0.26	
T+1	Co-dominant	GG	71.1±5.1 (51)	GG *vs* GA (0.8648)
		GA	117.9±3.1 (40)	GG *vs* AA (0.0002)
		AA	25.1±8.4 (4)	GA *vs* AA (0.0014)
	Dominant	GG		(0.9587)
		GA+AA		
	Recessive	GG+GA		(<0.0001)
		AA		
	Variant allele frequency		0.25	
V4	Co-dominant	CC	106.8±4.8 (56)	CC *vs* CG (0.0035)
		CG	64.7±2.8 (36)	CC *vs* GG (0.0685)
		GG	43.1±9.7 (3)	CG *vs* GG (0.7016)
	Dominant	CC		(0.0039)
		CG+GG		
	Recessive	CC+CG		(0.0164)
		GG		
	Variant allele frequency		0.22	
S1	Co-dominant	GG	86.3±7.2 (84)	GG *vs* GA (<0.0001)

		GA	108±7.5 (11)	
		AA	None	
	Dominant	GG	N/A	
		GA+AA		
	Recessive	GG+GA	N/A	
		AA		
	Variant allele frequency		0.06	
S2	Co-dominant	CC	44.9±1.5 (25)	CC *vs* CG (0.0999)
		CG	110.7±4.5 (52)	CC *vs* GG (0.3291)
		GG	86.9±7.3 (18)	CG *vs* GG (0.8681)
	Dominant	CC		(0.0548)
		CG+GG		
	Recessive	CC+CG		(0.4979)
		GG		
	Variant allele frequency		0.46	
Q-1	Co-dominant	TT	80.5±8.6 (62)	TT *vs* TC (0.0451)
		TC	115±5.0 (26)	TT *vs* CC (0.0062)
		CC	62.1±2.5 (7)	TC *vs* CC (0.3635)
	Dominant	TT		(0.0685)
		CT+CC		
	Recessive	TT+CT		(0.008)
		CC		
	Variant allele frequency		0.21	

Supplementary Table S3. The association of *ADAM33* genetic variants with the IgE levels among allergic rhinitis patients

	**IgE level (average ± SE)**							
**Genotype**	**T1**	**T2**	**T+1**	**V4**	**S1**	**S2**	**F+1**	**Q-1**
**Wild**	16.18±17.91	16.54±18.02	16.94±18.62	21.35±26.10	21.08±24.81	17.54±19.57	19.37±20.91	20.61±24.52
**Heterozygous**	32.11±33.29	30.34±33.01	30.21±33.17	17.06±19.46	12.53±13.48	21.22±26.95	18.90±25.03	19.73±23.19
**Homozygous**	6.40±4.60	6.40±4.62	6.40±4.61	20.24±24.03	-	27.30±29.25	27.33±30.45	3.72
***P* value**	0.003*	0.01*	0.02*	0.73	0.26	0.38	0.47	0.78

"*" Statistical significance using X^2 test
